# Regulation of amino acid metabolism in *Aphis gossypii* parasitized by *Binodoxys communis*

**DOI:** 10.3389/fnut.2022.1006253

**Published:** 2022-09-29

**Authors:** Hui Xue, Yunyun Zhao, Li Wang, Xiangzhen Zhu, Kaixin Zhang, Dongyang Li, Jichao Ji, Lin Niu, Jinjie Cui, Junyu Luo, Xueke Gao

**Affiliations:** ^1^Zhengzhou Research Base, State Key Laboratory of Cotton Biology, School of Agricultural Sciences, Zhengzhou University, Zhengzhou, China; ^2^State Key Laboratory of Cotton Biology, Institute of Cotton Research, Chinese Academy of Agricultural Sciences, Anyang, China

**Keywords:** parasitoids, amino acids, cotton aphids, host-microbe interactions, host-parasitoid interactions

## Abstract

The vast majority of parasitoids are capable of precise and meticulous regulation of nutrition and metabolism within the host. An important building block of life, amino acids are critical to the development of parasitoids. To date, research on how parasitoids regulate host amino acid metabolism remains limited. In this study, *Aphis gossypii* and its dominant parasitoid *Binodoxys communis* were used as a study system to explore how parasitism may change the regulation of amino acids in *A. gossypii* with UHPLC-MS/MS and RT-qPCR techniques. Here, for the first 8 h of parasitism the abundance of almost all amino acids in cotton aphids increased, and after 16 h most of the amino acids decreased. An amino acid of parasitic syndrome, the content of Tyr increased gradually after being parasitized. The expression of genes related to amino acid metabolism increased significantly in early stages of parasitism and then significantly decreased gradually. At the same time, the abundance of *Buchnera*, a cotton aphid specific symbiont increased significantly. Our comprehensive analyses reveal impacts of *B. communis* on the amino acid regulatory network in cotton aphid from three aspects: amino acid metabolism, gene expression, and bacterial symbionts. Therefore, this research provides an important theoretical basis for parasitoid nutritional regulation in host, which is highly significant as it may inform the artificial reproduction of parasitoids and the biological control of insect pests.

## Introduction

The regulation of host physiological mechanisms by parasitism is a highly complex and refined process which has evolved for thousands and tens of thousands of years. Parasites precisely control host metabolism so that the nutritional environment in the host is more suitable for the growth, development, and nutritional needs of the parasite (1–3). Although the phenomenon of parasitic insects changing host nutritional homeostasis is common (3, 4), its regulatory pathways and mechanisms remain largely unknown.

Parasitic insects, especially parasitic wasps, are representative models for the study of parasitic regulation. The parasitic characteristics of parasitic wasp larvae are very prominent in the regulation of host nutrition. Due to the parasitic characteristics and long-term development and evolution of parasitic wasps, a strong dependence on the host has evolved (5, 6). Normally, parasitic wasps mainly feed on the host’s hemolymph in early stages, and directly feed on the host’s body fat in later stages (7, 8). Studies have shown that the fatty acid composition of the parasitic wasp is very similar to that of the host (9), which may be due to parasitoids lacking an enzyme system for fatty acid synthesis. Therefore in order to meet their physiological and metabolic requirements, parasitic wasps directly use the fatty acids of the host (10). *Lysiphlebia japonica* directly increases the triglyceride content of cotton aphids to meet the growth and development of cotton aphid larvae by increasing the expression of genes related to the tricarboxylic acid cycle and glycolysis pathway in cotton aphid (11, 12). Furthermore, carbohydrates are important energy stores for organisms, and thus important substances for parasitic wasps to regulate in their hosts. Transcriptome analysis showed that after *Microplitis mediator* parasitized *Helicoverpa armigera*, more than 30 genes related to nutrition metabolism pathway were differentially expressed in the hemolymph of *H. armigera*, of which 20 genes related to glucose metabolism were significantly enriched (13).

Lipids and carbohydrates, as important energy-supplying substances for organisms, are crucial to the development of parasitic wasps, and similarly amino acids, as globally recognized nutritional small-molecules to life, are indispensable and important regulatory substances for mammals (14–18), plants (19, 20), and insects (21). Amino acids are traditionally classified into nutritionally essential (EAA) and non-essential (NEAA) categories according to animal growth or nitrogen balance (22), and play important roles in regulating animal food intake, nutrient metabolism, gene expression, and cell signaling (23–26). It has been reported that parasitoid wasps have a strong dependence on host amino acids due to their lack of some amino acid synthesis functions (27). Aphids are a special example of amino acid metabolism. Aphids feed on plant sap, but this sap is a poor diet and lacks certain essential amino acids, but bacterial symbionts (*Buchnera)* in host cells synthesize these amino acids providing them for the host (28–30). In this study, *Aphis gossypii* and its dominant parasitic wasp *Binodoxys communis* were used as the research system. Exploring the synergistic regulation mechanisms of parasite-host-symbiotic bacteria based on the specific amino acid metabolism patterns of parasitoid parasitism and aphids is both valuable and interesting from ecological, evolutionary, and agricultural perspectives.

At present, there are few studies on the regulation of host amino acid metabolism by parasitoids. The control mechanisms of parasitoids on host amino acid metabolism is remains unknown and may have powerful implications in the field in regards to fundamental questions in ecology and evolution as well as pest regulation. In this study, UHPLC-MS/MS and RT-qPCR techniques were used to detect changes in host amino acid content, related regulatory genes, and host symbiotic bacteria abundances under parasitic conditions to reveal the regulatory patterns and potential regulation mechanisms of parasitoids on host cotton aphids, thus building an important theoretical foundation for how parasitoids affect host metabolism.

## Materials and methods

### Insects and parasitoids

With *A. gossypii* and *B. communis* as the model research system, *A. gossypii* reproduced by parthenogenesis, and *B. communis* took cotton aphid as the host. Aphids were reared on cotton leaves at 26 ± 1°C and 65 ± 5% relative humidity with a 14:10 h light: dark photoperiod. *B. communis* were reared in laboratory on cotton aphids at 26 ± 1°C with a 14:10 h light: dark photoperiod and 65 ± 5% relative humidity. In order to obtain parasitized cotton aphids, 2-day-old cotton aphids were exposed to the *B. communis* environment, and parasitized cotton aphids were transferred to a *B. communis*-free environment after parasitism behavior was observed. In addition, the parasitoid wasps used in the experiment were all mated, and there were 10 female and male parasitoids in each parasitoid environment.

### Sample collection

The samples used for amino acid extraction and RT-qPCR in this study were the cotton aphid bodies parasitized by *B. communis* for 8, 16 h, 1, 2, and 3 day, respectively. The cotton aphid samples that were not parasitized but had the same growth and development were used as the control group. In a sterile environment, the cotton aphids parasitized for 16 h, 1, 2, and 3 day were manually dissected to remove the parasitic wasp larvae, and the control group was also subjected to the same corresponding dissection test. Three biological replicates were set for each stage of treatment and control samples. Cotton aphid samples were snap-frozen in liquid nitrogen immediately after dissection and stored at −80°C.

### Amino acid extraction and analysis

Samples were weighed (20 mg) and transferred to Eppendorf tubes. After the addition of two small steel balls and 250 μL of extraction solution (precooled at −20^°^C, acetonitrile-methanol-water, 2:2:1, containing isotopically-labeled internal standard mixture), the samples were vortexed for 30 s, homogenized at 40 Hz for 4 min, and sonicated for 5 min in ice-water bath. The homogenate and sonicate cycle was repeated 3 times, followed by incubation at −40^°^C for 1 h and centrifugation at 12,000 rpm (RCF = 13,800 (× g), R = 8.6 cm) and 4^°^C for 15 min. The supernatant was diluted 4 times, vortexed for 30 s to mix, and then transferred to an auto-sampler vial for UHPLC-MS/MS analysis.

The UHPLC separation was carried out using an Agilent 1290 Infinity II series UHPLC System (Agilent Technologies), equipped with a Waters ACQUITY UPLC BEH Amide column (100 × 2.1 mm, 1.7 μm). The mobile phase A was 1% formic acid in water, and the mobile phase B was 1% formic acid in acetonitrile. The column temperature was set at 35^°^C. The auto-sampler temperature was set at 4^°^C and the injection volume was 1 μL.

An Agilent 6,460 triple quadrupole mass spectrometer (Agilent Technologies), equipped with an AJS electrospray ionization (AJS-ESI) interface, was used for assay development. Standard ion source parameters were: capillary voltage = + 4,000/-3500 V, Nozzle Voltage = + 500/-500 V, gas (N2) temperature = 300^°^C, gas (N2) flow = 5 L/min, sheath gas (N2) temperature = 250^°^C, sheath gas flow = 11 L/min, nebulizer = 45 psi. The MRM parameters for each of the targeted analytes were optimized using flow injection analysis, by injecting the standard solutions of the individual analytes, into the API source of the mass spectrometer. Several of the most sensitive transitions were used in the MRM scan mode to optimize the collision energy for each Q1/Q3 pair. Among the optimized MRM transitions per analyte, the Q1/Q3 pairs with the highest sensitivity and selectivity were selected as the “quantifier” for quantitative monitoring. The additional transitions acted as “qualifiers” for the purpose of verifying the identity of the target analytes. Agilent MassHunter Work Station Software (B.08.00, Agilent Technologies) was employed for MRM data acquisition and processing.

A single peak was filtered to retain only peak area data with no more than 50% null in a single group or no more than 50% in all groups. The sorted data were analyzed by PCA (principal component analysis) and OPLS-DA (orthogonal projections to latent structures-discriminant analysis) using SIMCA (V16.0.2, Sartorius Stedim Data Analytics AB, Umea, Sweden). Differences between treatment means were statistically analyzed in SPSS for Windows (SPSS 20.0, Chicago, IL, USA) with the Student’s *t*-test.

### Nucleic acid extraction and cDNA synthesis

The samples were soaked in 75% ethanol for 30 s and then rinsed 3 times with ddH_2_O. DNA was extracted using the Fast DNA Spin Kit for SOIL (MP Biomedicals, Santa Ana, CA, USA). The concentration and purity of extracted DNA were detected by NanoDrop 2000C (Thermo Fisher Scientific, USA), and the DNA quality was measured with 1.5% agarose gel electrophoresis. Total RNA was isolated from samples using the TRIZOL total RNA isolation system (Invitrogen, Carlsbad, CA, USA) according to the manufacturer’s protocol. RNA quality was measured on 1.5% agarose gels and quantified using a Nano-Drop 2000 (Thermo Scientific, Wilmington, DE, USA). cDNA was synthesized from 1 μg of total RNA using the PrimeScript RT Reagent Kit with gDNA Eraser (Takara, Dalian, China), according to the manufacturer’s instructions. cDNA was used for RT-qPCR assay and DNA was used to detect bacterial copy number.

### RT-qPCR detection of gene expression

RT-qPCR was performed with the StepOnePlus™ Real-Time PCR System (Applied Biosystems, Foster City, CA, USA) using TransStart^®^ Top Green qPCR SuperMix (Yeasen Biotech, Shanghai, China) in 10 μl reaction volume. And the protocol was performed: 95°C for 3 min followed 40 cycles of 95°C for 5 s and 60°C for 30 s. *Dimethyladenosine transferase* (GenBank: KM507111) and *peptidyl-prolyl cis-trans isomerase* (GenBank: KF018924) were used as reference genes for gene expression normalization (11). Related gene-specific primers are shown in Supplementary Table 1. Gene expression data were analyzed using the 2^–ΔΔ^ ^Ct^ method (31). Differences between treatment means were statistically analyzed in SPSS for Windows (SPSS 20.0, Chicago, IL, USA) with the Student’s *t*-test.

### Quantification of bacterial communities

To estimate the *Buchnera* copy number, “absolute” real-time qPCR was conducted on DNA extracted from parasitized and non-parasitized aphids at 8, 16 h, 1, 2, and 3 day after parasitism (three biological replicates per treatment). The copy number of the target gene (F:5′-AGTATCGTAGAGGGAGGTA-3′, R:5′-CTTTCGCCACAGGTATTC-3′) was calculated according to the standard curve after dilution of the target gene sequence cloned in a Peasy-T1 cloning vector (TransGen Biotech, China). Using the StepOnePlus™ Real-Time PCR System (Applied Biosystems, Foster City, CA, USA) the following protocol was performed: 95°C for 3 min followed 40 cycles of 95°C for 5 s and 60°C for 30 s. Working in 10 μl volume using TransStart^®^ Top Green qPCR SuperMix (Yeasen Biotech, Shanghai, China). The corresponding standard curve for each reaction plate was calculated accordingly and three technical replicates were used for each sample. Differences in copy number between samples were statistically analyzed with Student’s *t*-test using SPSS 20.0.

## Results

### Principal components analysis and orthogonal projections to latent structures-discriminant analysis model analysis of amino acid extraction quality

**FIGURE 1:**
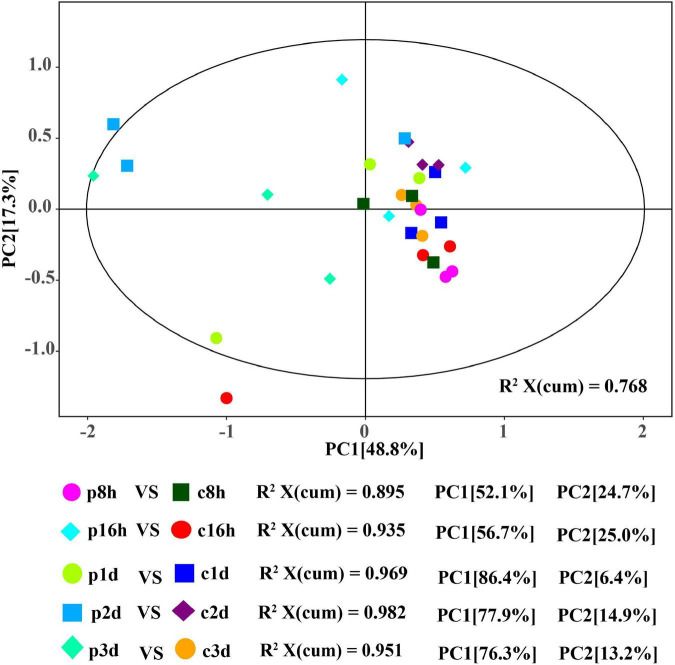
Score plot of the PCA models applied to parasitized and control aphids. p8h, p16h, p1d, p2d, and p3d indicate parasitized cotton aphids for 8, 16 h, 1, 2, and 3 days, respectively. Similarly, c8h, c16h, c1d, c2d, and c3d represent the above-treated control groups, respectively.

The quality parameters of PCA model R^2^X are all greater than 0.5 and the samples are basically in 95% confidence interval, therefore, the current model may be reliably used to characterize the metabolic differences between samples (Figure 1). Parasitized and unparasitized cotton aphids were visibly grouped separately in the same period, although there was a cross phenomenon in different developmental stages, but the amino acid metabolisms between the treatment (parasitized) and control (unparasitized) groups differed significantly (Figure 1).

In order to obtain more reliable information about the correlation between the differences in metabolites between control and treatment groups, orthogonal variables in amino acids that were not related to the classification variables by OPLS-DA analysis were filtered, and non-orthogonal variables and orthogonal variables were analyzed separately. The quality of the OPLS-DA model was tested by 7-fold cross validation. The R^2^X, R^2^Y, and Q^2^ of each comparison group were all greater than 0.739, 0.915, and 0.593, respectively (Table 1). The OPLS-DA score map can effectively distinguish the experimental group from the control groups (Supplementary Figures 1A–F), indicating that the current model can effectively distinguish samples from the parasitic and non-parasitic groups. The original model R^2^Y’s score approaches 1 through the permutation test for 200 random changes in the order of categorical variables, indicating that the established model conforms to the real situation of the sample data. The intercept of the regression line of Q^2^ and the vertical axis is less than 0. At the same time, with the gradual decrease in permutation retention, the proportion of the permuted Y variable increases, and the Q of the random model gradually decreases. Therefore, the original model is robust and there is no overfitting phenomenon (Supplementary Figures 1G–K).

**TABLE 1:** Cross-validation parameters of the OPLS-DA model.

Type	A	N	R^2^X (cum)	R^2^Y (cum)	Q^2^ (cum)	Title
OPLS-DA	1 + 1 + 0	6	0.739	0.915	0.593	p8h VS c8h
OPLS-DA	1 + 1 + 0	6	0.788	0.930	0.809	p16h VS c16h
OPLS-DA	1 + 1 + 0	6	0.774	0.967	0.843	p1d VS c1d
OPLS-DA	1 + 1 + 0	6	0.82	0.984	0.925	p2d VS c2d
OPLS-DA	1 + 1 + 0	6	0.767	0.917	0.687	p3d VS c3d

A, the number of principal components of the model; N, the number of samples in the model; R^2^X and R^2^Y, values indicate the total number of variations in the X and Y matrix explained by the model, respectively; Q^2^ represents the predictability of the models and relates to its statistical validity. Title: the data groups corresponding to the model.

### Parasitic regulation of essential amino acids in cotton aphids

**FIGURE 2:**
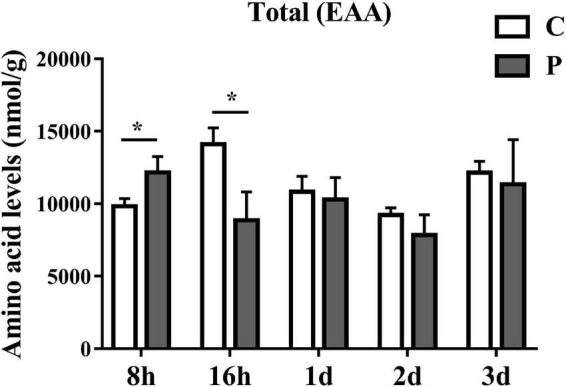
Parasitism causes changes in the content of total EAA (Essential Amino Acids) in cotton aphid. Statistical significance (Student’s *t*-test): *P < 0.05; **P < 0.01; ***P < 0.001.

**FIGURE 3:**
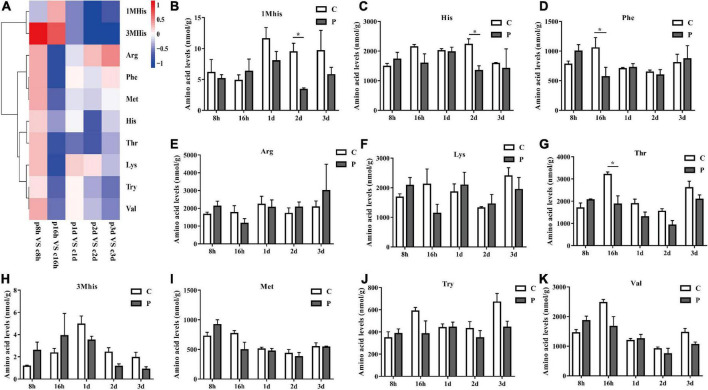
Parasitism leads to the change of EAA content of cotton aphid. Heatmap **(A)** and column **(B–K)** display of changes in EAA. 1Mhis, 1-Methyl-L-histidine; 3Mhis, 3-Methyl-L-histidine; C, Non-parasitized cotton aphids; P, Parasitized cotton aphids. The log2(Fold change) of amino acid content change is represented by heat map color scale, red indicates up-regulation and blue indicates down-regulation. Statistical significance (Student’s *t*-test): *P < 0.05; **P < 0.01; ***P < 0.001.

The content changes of all detected EAAs in each parasitic stage showed that the total EAAs of cotton aphids increased significantly at 8 h, began to decrease significantly at 16 h, and continued to trend downward 1–3 day (Figure 2). The heat map in Figure 3A summarize the log2(Fold change) in the essential amino acid content in cotton aphids in different stages of parasitism. The abundances of essential amino acids in cotton aphids were almost all greater at 8 h, and most of the essential amino acids showed a downward trend from 16 h (Figure 3). Only the abundance of the amino acid 1Mhis decreased in parasitized aphids at 8 h, and the decrease reached a significant level at the 2 day timepoint (Figure 3B). The amino acid His also decreased significantly in the 2 day timepoint (Figure 3C). Furthermore, the content of amino acid Phe decreased significantly in the 16 h timepoint, but showed an upward trend in the 3 day timepoint (Figure 3D). The overall analysis showed that only the quantities of Arg and Lys were significantly increased in different stages of parasitism (Figures 3E,F). Thr decreased significantly at 16 h (Figure 3G). Changes in 3His, Met, Try and Val did not reach significant levels (Figures 3H–K).

### Parasitic regulation of non-essential amino acids in cotton aphid

**FIGURE 4:**
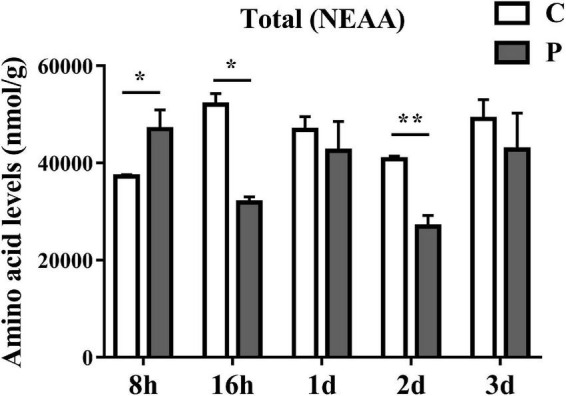
Parasitism causes changes in the content of total NEAA (Non-Essential Amino Acids) in cotton aphid. C, Non-parasitized cotton aphids; P, Parasitized cotton aphids. Statistical significance (Student’s *t*-test): *P < 0.05; **P < 0.01; ***P < 0.001.

**FIGURE 5:**
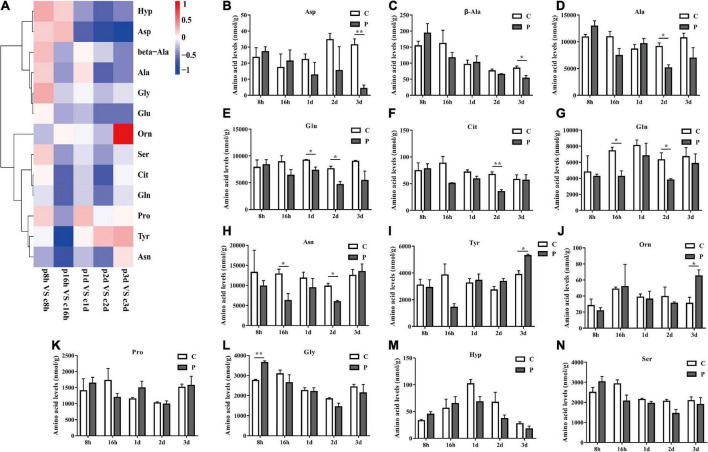
Parasitism leads to changes in NEAA content of cotton aphid. Heatmap **(A)** and column **(B–N)** display of changes in NEAA. C, Non-parasitized cotton aphids; P, Parasitized cotton aphids. The log2(Fold change) of amino acid content change is represented by heat map color scale, red indicates up-regulation and blue indicates down-regulation. Statistical significance (Student’s *t*-test): *P < 0.05; **P < 0.01; ***P < 0.001.

The overall trends in changes of total non-essential amino acids (NEAAs) content were similar to that of EAAs. Parasitism led to a significant increase in total NEAAs content at 8 h and a significant decrease at 16 h (Figure 4). The overall NEAAs trends in abundance are clearly visualized through heat map analysis (Figure 5A). The contents of amino acids Asp, β-Ala, Ala, Glu, Cit, Gln, and Asn decreased significantly (Figures 5B–H), but the content of Tyr increased significantly with as length of parasitism increased (Figure 5I). Tyr is also the key amino acid to reflect a parasitic effect. Orn increased 2.1 times significantly at 3 day (Figure 5J), and Pro content had an observable increasing trend but did not reach statistical significance (Figure 5K). Gly increased significantly at 8 h (Figure 5L), while Hyp and Ser had no significant change (Figures 5M,N).

### Parasitic regulation of amino acid metabolism gene expression

**FIGURE 6:**
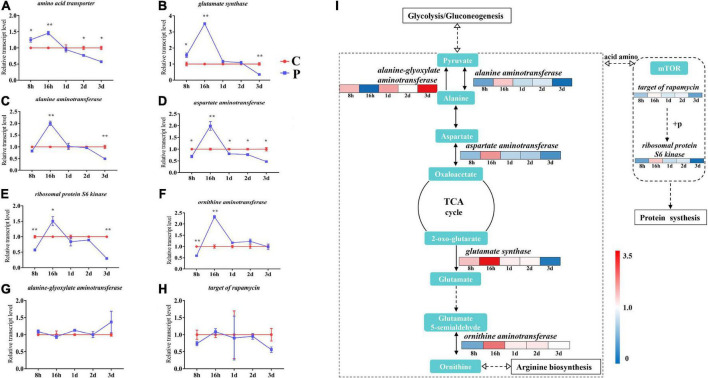
Changes in expression of amino acid metabolism genes in cotton aphid. Line chart **(A–B)** and signaling pathway **(I)** display of gene expression. C, Non-parasitized cotton aphids; P, Parasitized cotton aphids. The fold change in gene expression in the pathway is represented by the color scale, red indicates up-regulation and blue indicates down-regulation. Statistical significance (Student’s *t*-test): *P < 0.05; **P < 0.01; ***P < 0.001.

According to the difference of amino acid metabolism level caused by parasitism, we detected 8 key genes related to amino acid metabolism. The expression of *amino acid transporter* and *glutamate synthase* genes increased by 1.25 and 1.55 times at 8 h, 1.46 times and 3.51 times at 16 h, decreased after 16 h, and reached statistically significant levels at 3 day (Figures 6A,B). The expression levels of *alanine aminotransferase*, *aspartate aminotransferase*, and *ribosomal protein S6 kinase* genes were consistent, all decreasing at 8 h and increasing significantly at 16 h, and decreasing to a significant level at 3 day (Figures 6C–E). There was no significant difference in the expression levels of *ornithine aminotransferase* between 1 and 3 day, but the expression also significantly increased at 16 h (Figure 6F). There was no significant change in the expression levels of *alanine-glyoxylate aminotransferase* and *target of rapamycin* (Figures 6G,H). We grouped the aforementioned genes into metabolic pathways, including a heatmap representation of the fold change in gene expression, clearly showing the regulatory relationships between amino acid metabolism and synthesis, as well as the changes in pathway genes that utilize amino acids for protein synthesis (Figure 6I).

### Parasitic change of *Buchnera* abundance in *Aphis gossypii*

**FIGURE 7:**
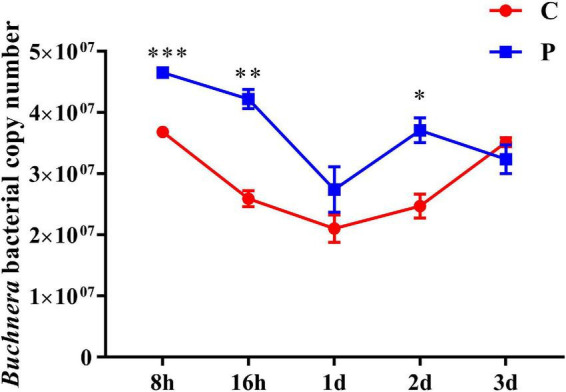
Changes in abundance of symbiotic bacteria *Buchnera* in cotton aphid. C, Non-parasitized cotton aphids; P, Parasitized cotton aphids. Statistical significance (Student’s *t*-test): *P < 0.05; **P < 0.01; ***P < 0.001.

As an obligate symbiotic bacteria in cotton aphid, *Buchnera* plays a key role in regulating the supply of amino acids. We detected the changes in the abundance of *Buchnera* bacteria measured with on copy number in cotton aphids at different stages of parasitism, and found that the abundance of *Buchnera* increased significantly at 8, 16 h and 2 day, which increased by 1.26, 1.63, and 1.50 times, respectively. During the 1 day period, although the difference of *Buchnera* abundance was not significant, it increased 1.3 times (Figure 7).

## Discussion

Parasites are ubiquitous in nature, and nutrient utilization is one of the most important strategies of parasitism (32). Parasitoid wasps create suitable host nutritional adaptations by regulating host nutrient metabolism (32). Amino acids are important nutrients for organisms, and understanding the regulation of parasitoids’ amino acid metabolism in the host is helpful to deeply understand the nutritional requirements of parasitoids, and is of great significance to the breeding and biological control of parasitoids. In this study, the amino acid content of parasitized and unparasitized cotton aphids at different developmental stages were analyzed with PCA and OPLS-DA. The differences between these samples were clearly distinguished, indicating that parasitism significantly affects the amino acid metabolism of cotton aphids, and that *B. communis* has a certain regulatory effects on the nutrition of cotton aphids.

Previous *in vitro* studies have demonstrated that the host provides some amino acids that are of considerable importance for the nutritional and metabolic adaptation of parasitoid wasps (33, 34). For example, there is currently evidence that the larval stage of *Aphidius ervi* is able to directly ingest the host’s sugars and amino acids through the body surface and gut. These nutrients are efficiently absorbed by the larval epidermis, but the rate of transport gradually decreases over the course of parasitization (33). With the decrease in the amino acid transport rate to the parasitoid wasp, the activity level of the host amino acid transport gene was likewise lowered, which was also confirmed by our findings here with *B. communis*, another endoparasitic wasp. In this study, the content of almost all amino acids in cotton aphids parasitized for 8 h increased, and this temporary increase was accompanied by a decrease in amino acid content. The most direct explanation for the change of amino acid content is related to the rate of parasitoid nutrient absorption and the activity of absorbed proteins (34). *B. communis* in the earliest stage of development at 8 h has no ability to absorb substances and lacks a digestive tract (35). *B. communis* has a faster rate of amino acid absorption in the early stage of larval development, for example, the content of some amino acids is greatly reduced at 16 h after parasitization. This indicates that parasitic wasp larvae begin to absorb or feed on some amino acids for physiological metabolism, and that the rate of absorption may be greater than cotton aphid’s ability to synthesize these molecules. The content of 1Mhis, Phe, His, Thr, Ala, Glu, Cit, and Gln decreased significantly from 16 h to 2 day, but there was no statistically significant difference in the later stages of development. Similarly, previous studies have shown that parasitoid larvae absorption rates of amino acids decrease in the later stage of development (33). Perhaps *B. communis* needs some amino acids to supplement in the later stage of growth and development. In regards to the nutritional regulation of parasitic wasps on the host, the TOR (target of rapamycin)-S6K (ribosomal protein S6 kinase) pathway was found to be inhibited, and the transcriptional translation process of cotton aphid may be decreased (36), which could meet the needs of parasitoids by reducing the consumption of amino acids (27).

As an amino acid with late and statistically significant parasitic effects, tyrosine is indispensable for the growth and development of endoparasitoid larvae (37). Tyrosine is an aromatic amino acid, which is synthesized in large quantities by phenylalanine through an aromatic shuttle mediated by symbiotic bacteria (38). Phenylalanine, as the only derivative precursor of tyrosine, decreased significantly at 16 h in our study, probably because tyrosine also decreased significantly at 16 h. In order to supplement the supply of tyrosine, a large amount of phenylalanine is needed to be involved in the formation of tyrosine. Another explanation is that the immune response of insects to biological attacks involves the extensive use of tyrosine through the phenoloxidase pathway (39), which may also be a means for parasitic wasps to regulate host tyrosine content. At present, the lack of lipid synthesis ability of parasitoid wasps has always been the focus of current research (40, 41). The N-acetylaspartate (NAA) pathway, which is involved in the production of aspartic acid, forms acetic acid, which is the only prerequisite for lipid synthesis (42). Furthermore, the NAA pathway is also an important regulatory pathway for lipid metabolism (43, 44). The change of aspartate content of cotton aphid was consistent with the trend in *aspartate aminotransferase* gene abundance. At the same time, aspartate aminotransferase is a part of malate-aspartate shuttle in human body, which is involved in gluconeogenesis in liver, kidney and glycerol in adipose tissue (45), which therefore suggests that parasitoids may use aspartic acid as an important regulator of lipid synthesis and thus regulate the lipid metabolism of cotton aphids. Based on this hypothesis, more verification experiments are needed to prove it.

Previously, Falabella et al. (37) proposed that bacterial endosymbionts affect the early growth and development of parasitic wasp larvae in aphids, and that the growth and reproduction of aphids depends on the obligate symbiotic bacterium *Buchnera* which provides essential amino acids (46, 47). Interestingly, parasitic wasps do not let go of the regulation of every detail of the host in order to satisfy their own growth and development. Other studies have proposed that parasitoids can alter the host’s microbial environment to increase favorable conditions for their colonization, and that parasitoids can adapt and even modulate the host’s microbial composition (48). Our results confirm the previous evidence that the parasitism of *B. communis* leads to an increase in the abundance of *Buchnera* in cotton aphids, which likely further supplements the biosynthesis of carbohydrates, lipids, and coenzymes in parasitic wasps by ensuring a steady supply of amino acids from the host (49, 50). Studies have shown that the symbiotic bacterial composition of parasitic wasp larvae is similar to that of host aphids, and that in particular the abundance of *Buchnera* is higher in parasitic wasp larvae (49). The shifts in *Buchnera* abundance in cotton aphid were no longer statistically significant in late stages of parasitization, perhaps in part because the bacterial composition of the parasitoid wasp was highly similar to that of the host. The *Buchnera* symbiont in the cotton aphid in the early stages of parasitism is most likely acquired by the parasitoid, and the parasitoid’s dependence on the host *Buchnera* gradually decreases in the later stage of development. Further investigation and experimentation is needed to confirm this hypothesis.

Parasitic wasps, as important biological controls natural enemy insects of pests, are critical to pest management (51). This research further clarifies the nutritional regulation and nutritional requirements of parasitoids, providing a strong theoretical basis for practical and inexpensive management of pests through the large-scale artificial reproduction of parasitoids.

## Conclusion

*Binodoxys communis* has a significant effect on amino acid metabolism of *A. gossypii*, involving associated gene expression changes and changes in abundance of symbionts associated with amino acid metabolism. This study provides an important theoretical basis for parasitic wasps to regulate the metabolism of the host *A. gossypii*, and provides a key reference for guiding parasitoid breeding and biological control of pests.

## Data availability statement

The data of this study can be provided directly by the corresponding author for further inquiries.

## Author contributions

JC, JL, and XG conceived and designed the research. HX conducted the experiments and wrote the manuscript. YZ, LW, DL, XZ, and KZ contributed the new reagents and analytical tools. HX, YZ, JJ, and LN analyzed the data. All authors contributed to the study conception and design, read and approved the manuscript.

## Abbreviations

PCA: principal components analysis
OPLS-DA: orthogonal projections to latent structures-discriminant analysis
1Mhis: 1-Methyl-L-histidine
3Mhis: 3-Methyl-L-histidine
Phe: Phenylalanine
Arg: Arginine
His: Histidine
Met: Methionine
Thr: Threonine
Try: Tryptophan
Val: Valine
Lys: Lysine
Hyp: 4-Hydroxyproline
β -Ala: beta-Alanine
Gly: Glycine
Ala: Alanine
Asp: Aspartic acid
Cit: Citrulline
Glu: Glutamic acid
Gln: Glutamine
Orn: Ornithine
Pro: Proline
Ser: Serine
Tyr: Tyrosine
Asn: Asparagine.
