# Supplementary material for: Regulation of amino acid metabolism in Aphis gossypii parasitized by Binodoxys communis
Source: Front Nutr. 2022 Sep 29;9:1006253. doi: 10.3389/fnut.2022.1006253 (PMC9558109; doi:10.3389/fnut.2022.1006253)
Supplement: Supplementary file 1 [file Data_Sheet_1.docx]

Supplementary Material


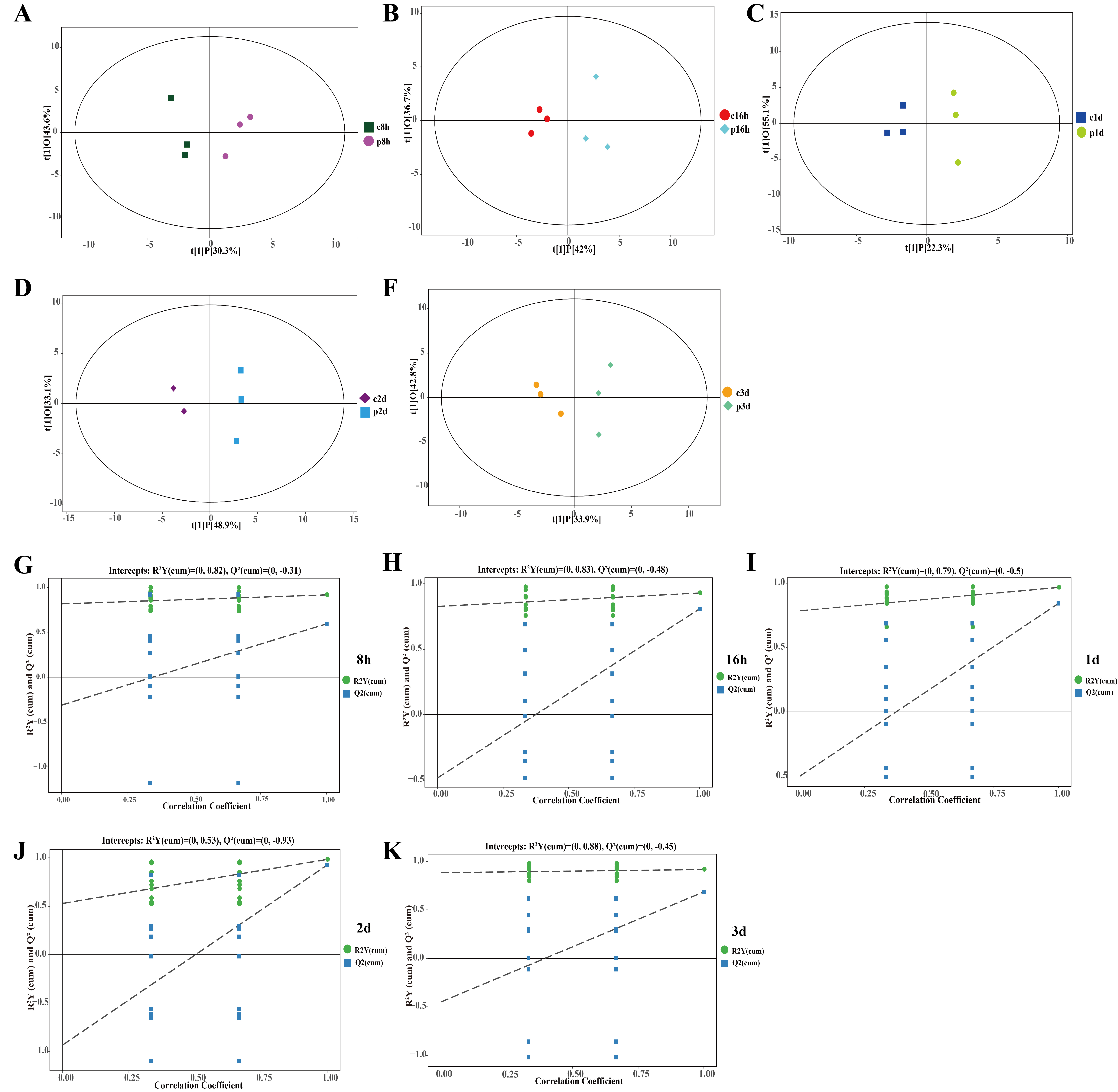


**Supplementary Figure 1.** OPLS-DA score plots and OPLS-DA permutation plots applied to parasitized and control aphids.

**Supplementary Table S1** Specific primers used in RT-qPCR.

| Genes | Primer sequence (5’ → 3’) | Efficiency | R^2^ |
| --- | --- | --- | --- |
| Amino acid transporter | F: GCCGAGTTCGGTGGCTACAA  R: AGTCGGATATTGGACAGTCCG | 1.13 | 0.993 |
| Glutamate synthase | F: GCTCGCTGTCGCACACCATC  R: CGGTAAAGTACGCCCACAGC | 1.09 | 0.992 |
| Alanine aminotransferase | F: GCTGTCCGGGGACCGTTATT  R: GTCCCATTGCGTGACAATCACC | 1.12 | 0.996 |
| Aspartate aminotransferase | F: AGGTGCTGGTGCTTATAGGG  R: GGCCAACAGATTTTCAGCTTG | 0.97 | 0.990 |
| Ribosomal protein S6 kinase | F: CAAAGAGGGAATGTTCCACG  R: GCTGGGCACATGTTGATGCG | 1.11 | 0.988 |
| Ornithine aminotransferase | F: CTGGTGTTGAGGGAGGAGAGTC  R: CCGTGGGCAAATACAACTTGCG | 0.90 | 0.984 |
| Alanine-glyoxylate aminotransferase | F: GCCCATGTTGGCCACTGTCA  R: CATGCAGGAACCTACTGTTGGT | 0.91 | 0.972 |
| Target of rapamycin | F: GGCTGCTGCAGGGTTTCAAC  R: CTTCGTGGAATCCAACACCAG | 1.12 | 0.989 |
